# Neuropathology of stress

**DOI:** 10.1007/s00401-013-1223-5

**Published:** 2013-12-08

**Authors:** Paul J. Lucassen, Jens Pruessner, Nuno Sousa, Osborne F. X. Almeida, Anne Marie Van Dam, Grazyna Rajkowska, Dick F. Swaab, Boldizsár Czéh

**Affiliations:** 1 SILS-Center for Neuroscience, University of Amsterdam, Science Park 904, 1098 XH Amsterdam, The Netherlands; 2 Department of Psychiatry, Douglas Institute, McGill University, Montreal, QC Canada; 3 Life and Health Sciences Research Institute (ICVS), School of Health Sciences, University of Minho, Campus Gualtar, 4710-057 Braga, Portugal; 4 ICVS/3B’s-PT Government Associate Laboratory, Braga, Guimarães, Portugal; 5 Max Planck Institute of Psychiatry, Munich, Germany; 6 Department of Anatomy and Neurosciences, VU University Medical Center, Neuroscience Campus Amsterdam, Amsterdam, The Netherlands; 7 Department of Psychiatry and Human Behavior, University of Mississippi Medical Center, Jackson, MS USA; 8 Netherlands Institute for Neuroscience, An Institute of the Royal Netherlands Academy of Arts and Sciences, Amsterdam, The Netherlands; 9 Department of Laboratory Medicine, Faculty of Medicine, University of Pécs, Pécs, Hungary; 10 Szentágothai János Research Center, Neuroendocrinology Research Group, University of Pécs, Pécs, Hungary

**Keywords:** HPA axis, Hippocampus, Prefrontal cortex, Amygdala, Mood disorder, PTSD

## Abstract

Environmental challenges are part of daily life for any individual. In fact, stress appears to be increasingly present in our modern, and demanding, industrialized society. Virtually every aspect of our body and brain can be influenced by stress and although its effects are partly mediated by powerful corticosteroid hormones that target the nervous system, relatively little is known about when, and how, the effects of stress shift from being beneficial and protective to becoming deleterious. Decades of stress research have provided valuable insights into whether stress can directly induce dysfunction and/or pathological alterations, which elements of stress exposure are responsible, and which structural substrates are involved. Using a broad definition of pathology, we here review the “neuropathology of stress” and focus on structural consequences of stress exposure for different regions of the rodent, primate and human brain. We discuss cytoarchitectural, neuropathological and structural plasticity measures as well as more recent neuroimaging techniques that allow direct monitoring of the spatiotemporal effects of stress and the role of different CNS structures in the regulation of the hypothalamic–pituitary–adrenal axis in human brain. We focus on the hypothalamus, hippocampus, amygdala, nucleus accumbens, prefrontal and orbitofrontal cortex, key brain regions that not only modulate emotions and cognition but also the response to stress itself, and discuss disorders like depression, post-traumatic stress disorder, Cushing syndrome and dementia.

## Stress and the brain

### The concept of stress

Whenever an endogenous or exogenous challenge is perceived as unpleasant, aversive or threatening, a series of systems and processes is activated that generates a coordinated response to that particular challenge, or stressor. This so-called stress response, an integral part of any adaptive biological system, is conserved throughout evolution. It is particularly active when an individual’s homeostasis, well-being, overall health or survival is threatened. An unpleasant surprise, relational or financial problems, the loss of a loved one, bereavement, unpredictability, an acute threat, e.g., when faced with an animal predator, or with psychosocial demand in humans, can all initiate a stress response. The same is true for perturbations of a more biological nature, such as an energy crisis, physical injury, hemorrhage or inflammation.

According to the popular press, stress is ever present in our modern, performance oriented and demanding society [109]. Stress contributes to several disabilities worldwide and as such represents a severe economical burden. The WHO expects that mental disease, including stress-related disorders, will be the second leading cause of disabilities by 2020. In the US, job stress alone, e.g., has been estimated to cost several hundred billions of USD every year in absenteeism, turnover, diminished productivity and medical, legal and insurance costs.

Stress, however, is no single entity and several different types of stressors can be distinguished: stressful challenges can be acute (being confronted with a predator or giving an important oral presentation) or of a chronic nature (living in poverty or in a broken family). It may occur only once, or may rather take place in a repetitive manner, that can be anticipated. Conversely, stress can be unpredictable and uncontrollable, mild or severe, and occurring in or out of context, e.g., of a learning experience [93, 102]. In addition, how stress exposure is actually perceived by an individual varies greatly, as does the persistence of its consequences.

Importantly, physiological ‘stress’ responses also occur following rewarding, “positive” and appetitive stimuli (e.g., winning a competition, sexual activity). Although they are often not considered to be stressors in classic, generally “negative”, terms, the physiological responses elicited by them can be as large as those seen after more aversive stimuli [104]. For the purpose of definition, a stressor in the context of this review will refer to any environmental demand that exceeds the physiological regulatory capacity of an organism, in particular during situations of unpredictability and uncontrollability. They are characterized by the absence of an anticipatory response (unpredictable), or a reduced recovery (uncontrollable) of the neuroendocrine reactions to stress. Taken together, depending on the type of stressor, various signals converge to orchestrate an integrated stress response that ‘resets’ many peripheral and central processes and allow an individual to adapt to the changes in its environment and thus to restore homeostasis.

The physiological stress response can be divided into two different time domains with a very quick response and a delayed response. The first phase of the stress response is considered the “alarm reaction” or the “fight-fright-or-flight” response, which involves the rapid activation of the autonomic nervous system (ANS) that causes the release of epinephrine and norepinephrine from the adrenal medulla. These hormones quickly elevate basal metabolic rate, blood pressure and respiration, and increase blood flow to the more vital organs that are essential for the “fight-or-flight” response, such as the heart and skeletal muscles. At a later stage, the hypothalamic–pituitary–adrenal (HPA) axis is activated as well. In this classic neuroendocrine circuit, limbic and hypothalamic brain structures coordinate emotional, cognitive, neuroendocrine and autonomic inputs, which together determine the magnitude and specificity of an individual’s behavioral, neural and hormonal responses to stress.

**Fig. 1:**
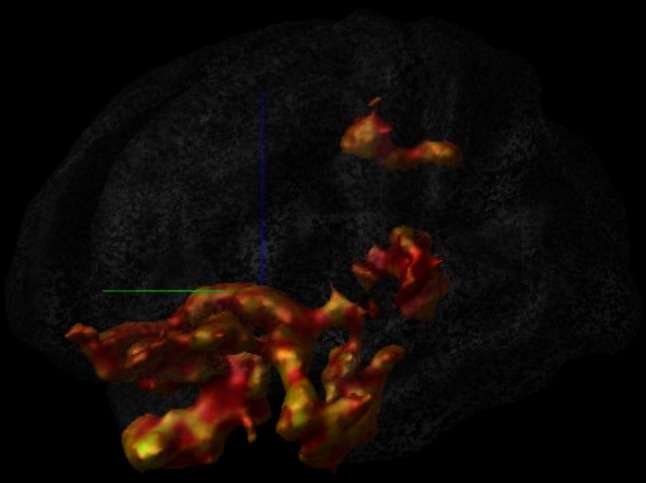
Identification of the brain regions that show activational changes during acute stress. Data are based on averages of 21 healthy subjects who were exposed to the Montreal Imaging Stress Task and showed subsequent cortisol increases, for details see [159]. Activation is prominent in a variety of limbic system and frontal lobe structures, including hippocampus, amygdala and the anterior cingulate cortex

This second response is mediated by glucocorticoid (GC) hormones (corticosterone in rodents and cortisol in humans) which generally act in a slow, genomic manner as transcriptional regulators of glucocorticoid responsive genes. Fast (non-genomic) GC actions have also been described and their actions are mediated by putative membrane-bound receptors. It should be emphasized that other signalling pathways act in concert with the HPA axis like the gonadal axis, the adipose axis, and the immune system. All these help to direct energy resources such that attention can be focused on the most urgent and important elements of the challenge while other, less urgent functions, e.g., food intake, digestion or reproduction, are temporarily suppressed [93]. The profound change in activational patterns that is induced by perceiving a situation as stressful can nowadays be visualized by functional magnetic resonance imaging (fMRI) studies, and as can be seen in Fig. 1, encompasses a variety of limbic system and frontal lobe structures, including hippocampus, amygdala and the anterior cingulate cortex. Throughout the text, we focus on the HPA axis, because this system is most heavily investigated, but it should be emphasized that several other systems contribute to the stress response as well (see “Inflammatory changes and depression”, “Gender differences in depression: relationship with HPA axis activity”, “The glutamatergic and GABAergic systems in depression”, “Glial plasticity”, “Water content, volume changes and altered vasculature”).

### HPA axis, stress hormones and their receptors

HPA axis activation is triggered by corticotropin-releasing hormone (CRH) in the paraventricular nucleus (PVN) that induces adrenocorticotropic hormone (ACTH) release from the pituitary, which in turn releases GCs from the adrenal. Regulation occurs through negative feedback after GC binding to high-affinity mineralocorticoid (MR) and lower affinity glucocorticoid receptors (GR) [47]. HPA axis activity is not only affected by stress, (see “Inflammatory changes and depression”) and a bi-directional communication exists, e.g., between the immune and neuroendocrine stress systems.

The GR helps to maintain GC levels within physiological limits [57, 104]. Aberrant GR expression has been implicated in stress resistance, anxiety and depression [47, 172, 234]. GC plasma levels are under circadian and ultradian control [115, 163]. Together, MR and GR determine sensitivity of the brain to stress [77, 135, 160, 203] and thereby modulate attention, vigilance, behavior and memory formation and eventually adaptation and coping with stress.

### Glucocorticoid hormone actions and hippocampal pathology; the ‘glucocorticoid cascade’ hypothesis

**Fig. 2:**
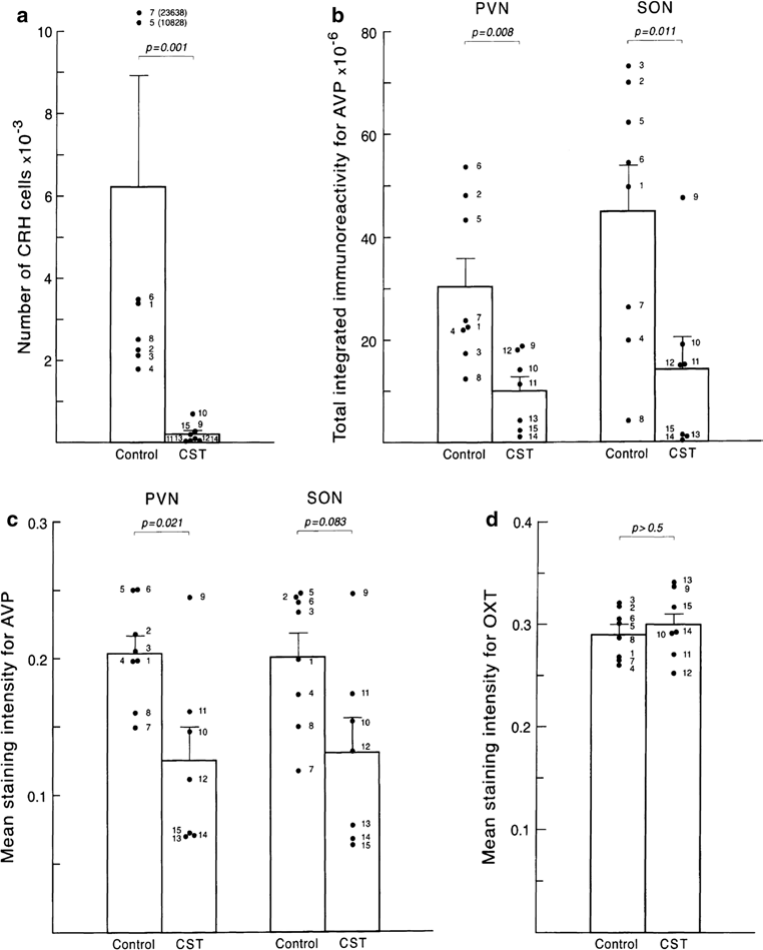
Exogenous or synthetic glucocorticoid treatment affects the human brain and reduces the numbers of CRH-immunoreactive cells in the hypothalamic PVN (**a**) but has no effect on oxytocin immunoreactivity in the PVN of corticosteroid-exposed subjects (**d**; CST). Reproduced, with permission, from [59]

Upon their release in the periphery, GCs enable an individual to engage in an adaptive response to a stressor by affecting energy and lipid metabolism, among others. The large numbers of GRs in the brain, and particularly the hippocampus, make it vulnerable to elevated GC levels [47, 120, 209]. The GR occurs in at least two isoforms (GRalpha and GRbeta), with the GRalpha isoform being the most predominant one in brain. In addition to rodent studies, [171] several human brain regions express GR and MR [77, 162, 189, 233]. In humans, considerable diversity of GR and MR transcripts exists [49, 100], which includes 13 exon 1 mRNA variants and 8 N-terminal variants, that arise from the GRalpha isoform. In the human hypothalamus, GRalpha protein is selectively expressed in CRH-containing parvocellular, but not magnocellular, neurons of the PVN [231]. Application of exogenous, synthetic GCs strongly suppresses CRH and vasopressin production in neurons of the human PVN (Fig. 2) [59], whereas oxytocin (OXT) neurons are not affected.

In contrast to the relative paucity of GR in the rhesus monkey [175], the human hippocampus shows abundant GR protein expression in CA1 and DG neurons, although the CA3 subregion has lower levels. GR is additionally expressed in astrocytes and overall GR levels remain stable with age. Increases have been found in the hippocampus and amygdala of patients with major depression (MDD) [229, 231, 232]. Thus, even though less is known about the MR [162], the prominent expression of GR in human brain makes these regions important targets for stress exposure [2, 93, 100]. Whereas acute and short-term stress is generally considered beneficial and adaptive, chronic stress may cause an MR/GR imbalance or down-regulation [47, 162] which can alter HPA feedback and result in overexposure of the brain and peripheral tissues to these powerful steroids.

When considering neuropathological aspects of stress, earlier animal experiments had reported brain damage after prolonged periods of stress, especially in aged animals and mainly in the hippocampus. For example, Landfield et al. [107] showed that cumulative GC exposure influenced hippocampal viability and compromised cognition. Subsequently, Sapolsky et al. [178, 182] reported that chronic stress causes a loss of pyramidal neurons in the hippocampus, accompanied by cognitive deficits in rats. Another study described that training rats for 6 months in a two-way shuttle escape task, using low intensity foot shock stress (4 h/days) resulted in endogenous hypercortisolism and CA1 pyramidal neuronal loss in senescent rats [98], while others reported reactive gliosis, reduced dendritic branching and reductions in volume and in CA1/3 cell numbers [181, 183].

The hippocampus was previously thought to inhibit CRH activity directly, and given its high MR and GR density [171, 189, 231], damage to the hippocampus was expected to cause a disinhibition of CRH activity, thus increasing the drive on the HPA axis, which in turn would further stimulate GC levels and aggravate hippocampal damage. This feed-forward “glucocorticoid cascade hypothesis” was proposed to be a pathogenic mechanism underlying stress effects on the brain, and considered relevant for human disorders associated with peripheral HPA axis changes that were paralleled by structural changes in the hippocampus, like Alzheimer’s disease (AD), post-traumatic stress disorder (PTSD), Cushing’s disease and depression [182].

These interpretations were supported by studies on subordinate, wild-born vervet monkeys that had experienced prolonged, severe social stress in captivity and that displayed post-mortem adrenal hypertrophy. Although the coincident hippocampal degeneration in these animals was consistent with the glucocorticoid cascade hypothesis, the morphological alterations and neuron loss in these, and also in other non-human primates implanted with cortisol pellets in their hippocampi [183], were most pronounced in CA neurons [217], brain regions particularly sensitive to pressure artifacts; after death, the soft tissue of the brain can be compressed by its own weight or by manipulation during brain collection, which refers to “post-mortem compression”.

While the initial studies had used rather extreme, physical stressors or extremely high pharmacological GC concentrations [101, 181], later studies in non-human primates and rodents that used more relevant psychosocial stressors [112, 219, 223], added conflicting data insofar that they did not observe massive neuronal loss or obvious neuropathology following chronic stress when this was assessed using unbiased stereological tools [157, 201, 223]. For example, when adult male rats were chronically treated with the selective GR agonist dexamethasone, with dexamethasone plus the selective MR agonist aldosterone, or with supraphysiological doses of corticosterone, only dexamethasone treatment reduced DG granule and CA3 pyramidal cell numbers [202]. However, this synthetic steroid differs from the endogenous corticosterone in terms of brain penetrance and retention and although it can induce cognitive deficits in aged mice [238], it did not influence the CA1 or hilar subfields. In contrast to dexamethasone, animals injected with corticosterone failed to reveal any change in cell number in any of the hippocampal subfields, although volume reductions in hilus and CA3 were observed [201]. Also in tree shrews exposed to chronic subordination stress, the CA1 and CA3 pyramidal neuron numbers were not different from controls, despite elevated cortisol levels [223]. Chronic studies in pigs, non-human primates and chickadees yielded similar results while changes in apoptosis were also not demonstrated [112, 157, 219].

It is now known that the tonic inhibitory control on HPA axis activity [82] is exerted through several, often indirect, neural pathways including the bed nucleus of the stria terminalis (BST), the amygdala and the endocannabinoid system [212, 221]. The GC-mediated negative feedback of the HPA axis thus takes place at several levels, including the hypothalamus and pituitary, and not only at the hippocampus [82, 84]. Also, although GCs target hippocampal cells, they can increase vulnerability to, but not directly cause, subsequent insults [33]. Moreover, an absence of massive structural changes after GC exposure does not necessarily imply that no functional, molecular or changes in responsivity [43] are induced. Furthermore, variables like genetic risk factors, early life programming and structural plasticity were not considered at the time. As a consequence, the GC cascade hypothesis has been refined and rephrased over time [31, 119, 134, 154, 160]. Together with the corticosteroid receptor hypothesis of depression [87] and the realization that GCs are on the other hand, essential for, e.g., dentate viability [197], it has sparked the development of drugs selectively targeting specific stress system components [88, 188, 243].

## Molecular, cellular and functional changes in stress-related psychiatric disorders

It is difficult to describe the complete and exact neuropathological consequences of stress exposure in the human brain as stress can occur in so many different forms and depends on an individuals coping strategy and stress sensitivity. Also, there is no single unique disease or direct neuropathological hallmark that is always, only and directly, caused by stress exposure per se. Notably, many of the stress-induced changes are plastic and reversible in nature, for this phenomena we use the term “neuroplasticity”, which refers to the general capacity of the brain to adapt functionally or structurally to a change in demands, and we further specify when particular forms of plasticity are discussed.

However, severe or prolonged stress is well known to increase the risk to develop psychopathologies such as PTSD, depression, schizophrenia or anxiety disorders in susceptible individuals and may even trigger psychotic episodes. Clear brain changes have been described in these conditions and our aim is to summarize those. We focus on disorders where the relationship between stress and the disease occurrence is well documented and where obvious structural alterations have been reported, like in depression, PTSD, Alzheimer’s and Cushing’s disease.

### Stress-related changes in major depressive disorder (MDD)

Stress is the most common risk factor for the development of mood disorders like MDD that are thought to result from interactions between genetic predispositions and the environment [173]. Especially stressful life events experienced during early childhood or adolescence can significantly increase the risk to develop depression [78]. Indeed, in a large proportion of depressed patients the HPA axis activation and GC feedback resistance is common. This is reflected by the high percentage of dexamethasone non-suppressors in this population as well as hypertrophy of the adrenals and pituitary and increased plasma levels of cortisol, particularly during the through of the circadian rhythm, and increases in CRH and AVP expression in the PVN [209]. Notably, depressed subjects show remarkable heterogeneity in neuroendocrine function and the proportion of depressed individuals demonstrating overt HPA axis abnormalities may range from 35 to 65 %.

#### Hypothalamic neuropeptide changes in depression

##### CRH in depression

**Fig. 3:**
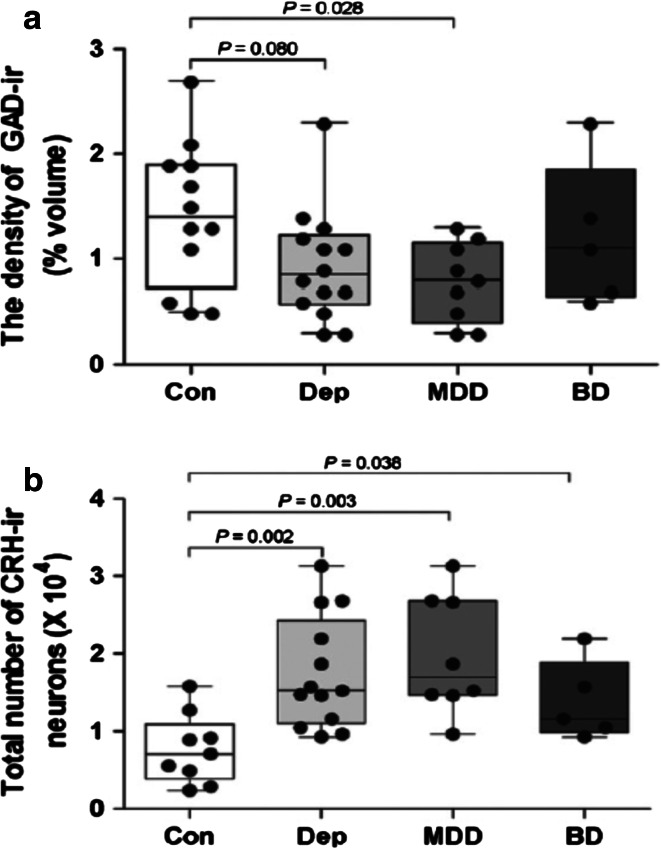
Quantification of glutamic acid decarboxylase (GAD)65/67-immunoreactivity (GAD-ir) (**a**), the total number of corticotropin-releasing hormone (CRH-ir) neurons in the hypothalamic paraventricular nucleus (PVN) (**b**) of depressed patients and controls. *Con* controls, *Dep* depression, *MDD* major depressive disorder, *BD* bipolar disorder. Significant increases are found in total numbers of CRH-ir in the depressed, major depressed and bipolar groups (**b**, see also [166]), whereas GAD65/67-ir is significantly reduced in the major depressed group (**a**). From [65], with permission

CRH-expressing neurons in the hypothalamic PVN are the central driving force of the HPA axis. The number of CRH expressing neurons, the number of CRH neurons co-expressing AVP, and the amount of CRH-mRNA in the PVN are significantly increased in depressed subjects, independent of whether they died during a depressive state or not (Fig. 3) [166]. Since intracerebroventricular injection of CRH induces symptoms of depression in rodent [87], centrally released CRH may be implicated in depression etiology. During post-natal development of the stress system, CRH controls HPA axis activity and mediates effects of early disturbances like maternal deprivation, through the CRH receptor CRH-R1. Both basic and clinical studies further suggest that disrupting CRH signaling through CRH-R1 ameliorates stress-related clinical conditions. Although CRH in CSF is also derived from other brain areas like the thalamus, CRH concentrations of healthy controls and depressed patients decrease after treatment with antidepressant drugs [83]. Lastly, in depressed patients the significantly increased CRH-mRNA levels in the PVN are accompanied by an increased expression of genes involved in CRH activation, such as CRH-R1, MR, estrogen receptor-alpha (ER-α) and AVPR1a, and with a significantly decreased expression of genes involved in the inhibition of CRH neurons, such as the androgen receptor (AR) mRNA [8, 228]. These findings [228] raise the possibility that a disturbed receptor balance in the PVN contributes to a CRH-mediated HPA axis activation in depression.

##### Arginine vasopressin (AVP), stress and depression

There are at least four different vasopressinergic systems intimately involved in the signs and symptoms of depression [209]. First, AVP is produced by the magnocellular neurons of the hypothalamic SON and PVN, whose axons run to the neurohypophysis where they release AVP and OXT into the general circulation. Circulating AVP targets the anterior pituitary while high levels also affect mood. Secondly, the parvocellular neurons of the PVN secrete CRH and AVP also as neurohormones from their axons in the median eminence, into the portal capillaries that transport them to the anterior lobe of the pituitary. AVP strongly potentiates ACTH-releasing activity.

Third, vasopressinergic fibers project from the hypothalamus to subregions of the hippocampus, septum, amygdala and brainstem, where AVP serves as a neurotransmitter/neuromodulator via AVPR1a and AVPR1b receptors. Moreover, magnocellular neurons release AVP from their dendrites and somata to act as local neuromodulators on receptors close to their site of release. Fourth, AVP is released into the brain with a circadian rhythm by neurons of the biological clock or SCN, which shows significant changes in depression. Once overexpressed, AVP may contribute to hyper-anxiety and depression-like behaviors, whereas AVP deficits may, in addition to diabetes insipidus, cause signs of hypo-anxiety and disturbed rhythmicity [108].

A misbalance of multiple genes involved in HPA axis regulation may occur in the PVN in depression with a possible role for AVPR1a [228]. Since also early types of stress can epigenetically program the AVP gene in a long-lasting manner [139, 140], the AVP-driven HPA axis hyperactivity in depression is receiving more attention [136].

In the PVN of depressed patients, the number of AVP and OXT protein expressing neurons is increased while for AVP mRNA, a 60 % increase in expression was found in the SON in melancholic but not in non-melancholic depression [136]. Enhanced AVP mRNA production leads to increased plasma levels of AVP [220] that have been related to an enhanced suicide risk in depression, as well as to an anxious-retarded type of depression, psychomotor retardation and memory disturbances in depression.

#### Hippocampal changes in depressed patients

Maladaptive responses to stress and the associated GC hypersecretion can induce hyperemotional states, mood dysfunction and cognitive impairments in depressed patients. This is often paralleled by volume changes in various brain regions including the hippocampus. In early studies, considerable variation was found in hippocampal volume in depression [124, 160]. Several explanations for this variation have been put forward including the higher spatial resolution in the more recent studies or differences in disease duration [195], anatomical delineation [179], lateralization, early life conditions and genotype [22, 158, 160, 215], the presence of abuse [215] and/or pharmacological treatments [116]. Hippocampal volume reductions in depression are by now one of the best-replicated findings in biological psychiatry [97], but whether it is cause or consequence of the disorder remains unclear. Predictors of lower hippocampal volumes in patients were: a more extensive depressive episode duration and recurrence, the size of their integrated cortisol responses and a history of early life stress [46, 62, 160, 226], while a smaller hippocampal volume could also predispose for the development of psychopathology [180].

Although classic MRI studies generally demonstrated a lower volume of the entire hippocampus in depression, spatial resolution generally precluded in vivo measurement of distinct hippocampal subfields, even though preclinical and some post-mortem studies indicated that chronic stress and depression affect hippocampal subfields, and different structural substrates, to a different extent. In addition to subfields across its transversal axis, the hippocampus also shows topographical segregation along its longitudinal axis [61]. Improved spatial resolution of high field strength MRI has now enabled to identify connectivity changes [245] and detailed measurements of subfield areas [90, 92, 235]. They revealed that the mean volumes of the DG and CA1-3 subregion were smaller in non-medicated or recently unmedicated depressed patients than in healthy controls. Along the longitudinal axis, a smaller volume was mainly found posteriorly, i.e., in the hippocampal body and tail, rather than anteriorly in the hippocampal head [90].

Of interest, both the subfield and posterior hippocampal volume reductions were seen only in unmedicated depression but were absent in patients treated with antidepressants. The posterior hippocampus may thus be particularly susceptible to volume changes [130, 143, 184], the extent of which may even predict a worse treatment outcome [128]. Furthermore, successful long-term antidepressant treatment also seems to increase posterior hippocampal volume [184]. In agreement with preclinical studies of chronic stress, decreases in CA3 volumes were shown [90] that, based on rodent studies, it could be related to different structural substrates, as will be discussed below.

**Fig. 4:**
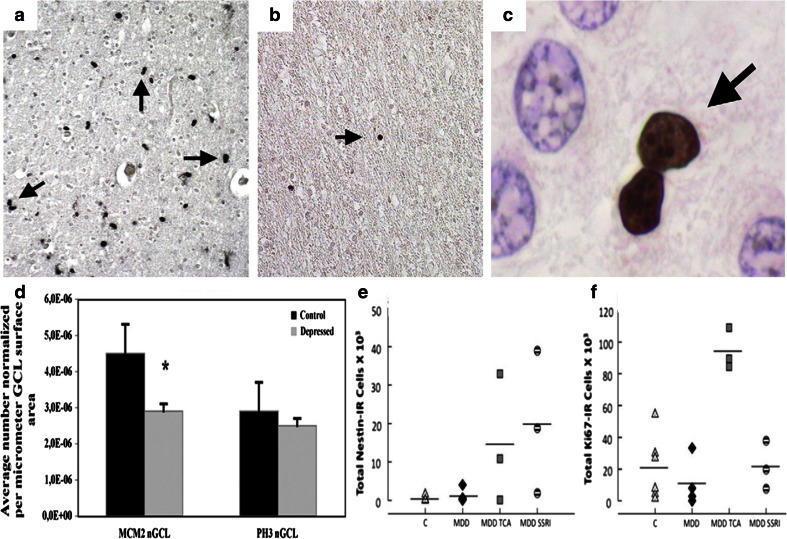
Changes in proliferation in the human brain of depressed and anti-depressant-treated patients. **a** Cells immunopositive for the cell cycle marker minichromosome maintenance protein 2 (MCM2) that is involved in the control of DNA replication. In the hippocampus, many MCM2-immunopositive cells and doublets (*arrows*) are observed in cortical tissue of a 2-year-old subject that served as positive control. **b** MCM2-ir cell numbers are strongly reduced to very low numbers (*arrow*) in a 69-year-old control subject. **c** MCM2-ir doublet of 2 still closely attached cells that appear to be about to separate in the hippocampus of a depressed patient (*arrow*), cresyl violet counterstain. Cells are viewed under a 10× magnification (**a**, **b**) or at 63× (**c**). **d** Graphs depicting numbers of MCM2 and phosphorylated histone H3 (PH3) immunopositive cells (the latter marker reflecting late G2 and mitotic phase of cell division). PH3 immunoreactive cells in the subgranular zone and granular cell layer of the dentate gyrus, normalized to the surface area of the GCL and expressed per square micrometer. A significant reduction is found for MCM2, but not PH3, in a cohort of 10 elderly (average age of 68 years) depressed patients compared to 10 controls. **e** Neural progenitor and **f** dividing cells (Nestin and Ki-67 as respective immunocytochemical markers) are increased in the dentate gyrus of a younger cohort of (average ages of 40 and 54 years) of patients with major depressive disorder (MDD) who were treated with antidepressants compared to untreated MDDs and control subjects. Progenitor numbers (**e**, Nestin-ir) were higher in MDD patients treated with tryciclics (TCA) or with selective serotonin reuptake inhibitors (SSRI), compared to untreated MDD and Control cases whereas the numbers of dividing cells (**f**, Ki-67-ir) were higher only in the TCA but not SSRI-treated group (*n* = 5–7 cases per subgroup). Reproduced, with permission, from [15, 121]. [121] was used for (**a**–**d**) and [15] was used for (**e**, **f**)

In a recent post-mortem study, hippocampal tissue of 17 pairs of MDD and control subjects, all around 50 years of age, were analyzed stereologically. While hippocampal volume in all MDD subjects was not significantly smaller compared to control subjects, total volume in MDD was decreased with duration of depressive illness. Also, there was no significant difference between MDD and controls in total number or density of the pyramidal neurons and granule cells or glial cells in the CA1, CA2/3, hilus, or DG subregion [29]. However, CA1 pyramidal neuron density increased with duration of illness in MDD and both granule cell and glial cell numbers increased with age in MDD patients on medication which may reflect proliferative effects of antidepressants (see Fig. 4, “Adult hippocampal neurogenesis”). This suggests that reductions in volume parallel to increased cellular densities are best explained by assuming cell shrinkage and hence changes in neuropil rather than cell loss [29, 206]. Also changes in water content may be implicated (see “Water content, volume changes and altered vasculature”).

Imaging studies in patients with stress-related affective and emotional disorders have shown that also the volume of structures other than the hippocampus like the PFC, cingulate cortex, hypothalamus and amygdala, as well as their interactions and coherence, is altered by stress [45, 147, 160, 200]. Finally, it should be noted that hippocampal shrinkage is not stress specific and has also been reported in several other brain disorders including schizophrenia, dementia, Parkinson’s and Huntington’s diseases, epilepsy and alcoholism [36].

#### Stress effects on the amygdala

While the hippocampus mediates spatiotemporal aspects of behavioral impairment, the amygdala contributes to the affective and emotional aspects of cognition [110]. Clinical studies have shown increases, decreases or no change in volume of the amygdala in MDD [23] and a meta-analysis even found no changes in amygdala volume in depressed patients although increases in cerebral blood flow to the amygdala, a correlate of neuronal activity were reported [54]. Recent experimental studies have identified cellular and molecular correlates of stress-induced amygdaloid plasticity that may underlie anxiety and depressive-like behavior. Animals exposed to chronic stress exhibited enhanced anxiety in the elevated plus-maze while at the cellular level, a persistent increase in dendritic arborization and higher spine density was found across the primary and secondary branches of the basolateral amygdala (BLA) spiny neurons and even spine formation was induced [137].

This dendritic hypertrophy in the BLA after stress is distinct from the retractions seen in the hippocampal CA3, which is reversible after a stress-free period [225]. In contrast, the BLA does not re-adjust morphologically or functionally after such a recovery period [137, 225]. This adds to accumulating evidence that structural encoding of aversive experiences, through enhanced availability of post-synaptic dendritic surface and synaptic inputs on principal neurons of the BLA, may facilitate symptoms of chronic anxiety and disorders like MDD and PTSD by enhancing synaptic connectivity in the BLA [154].

Such changes resemble findings in humans where larger amygdala volumes have been found, partly related to early stress exposure [125, 160]. The apparently conflicting clinical and basic findings could be due to heterogeneous clinical populations, where depression is comorbid with other psychiatric illness, or medication effects, as some patients were undergoing treatment during the investigations. At the same time, the stress paradigms used in basic research are incomplete models not capable of fully recapitulating the human disease state. Furthermore, most of the basic science findings were gathered from adult rodent stress and did not take into account early stress, which has been implicated in depression vulnerability [122].

#### The prefrontal cortex (PFC)

The PFC participates in cognitive, socio-emotional and executive functions that are sensitive to stress [4, 26, 40, 50]. Furthermore, the PFC modulates autonomic and neuroendocrine responses to stress [82, 151]. As the human PFC is one of the last regions to complete maturation, it contains neurons with more complex dendritic trees than earlier maturing cortical structures. This prolonged development makes the PFC susceptible to disruption and it indeed is affected in developmental neuropsychiatric disorders like autism and schizophrenia [214]. GRs and MRs are abundantly expressed by neurons and glia in the PFC of rodents and primates [175] and regulated by stress [153]. A recent human study investigated developmental changes in GR expression in the dorsolateral PFC from infancy to adulthood and found dynamic patterns of GR isoform expression across the lifespan, suggesting that the neonatal and late adolescent periods represent vulnerability windows to stress during human cortical development [191]. Furthermore, abnormal GR isoform expression levels have been found in the PFC of patients with psychiatric disorders like schizophrenia, bipolar and major depressive disorders [162, 190, 192]. Transcript level of MR was significantly decreased, while the ratio of GRα to MR mRNA was increased in the anterior cingulate cortex (ACC) and the dorsolateral PFC (DL–PFC) of depressed patients. Thus, a selective disturbance of MR and of GRα/MR ratio may exist in the ACC/DL–PFC in depression that is inversely correlated to the corresponding levels in the PVN and may thereby contribute to HPA axis hyperactivity and depression etiology [162].

A large body of evidence further demonstrates that repeated stressful experiences have a profound impact on neuronal plasticity in the PFC. The most thoroughly investigated neuromorphological change is the regression of the geometrical length of apical dendrites of pyramidal neurons in layers II–III of the mPFC [26, 40, 164]. The main results from these studies are a significant reduction in total dendritic length of 20–35 % with a significant decrease in branching and spine density of the distal apical dendrites. These chronic stress-induced effects are likely to be mediated partially by the activation of GRs and NMDA receptors [156] because artificially elevated levels of GCs result in morphological changes similar to those seen following chronic stress exposure [26, 40, 164], while blocking NMDA receptors could prevent these stress-induced effects [132]. It also appears that these changes are plastic and not degenerative in nature, because they reverse spontaneously after a recovery period [71, 165], at least when animals are young, as middle-aged and aged rats failed to show this reversible dendritic remodeling [13].

Notably, most rodent studies [26, 40, 164] report a significant impact of stress on the apical but not basal dendrites. However, the only comparable human study that examined dendritic branching of pyramidal neurons in the ACC of depressed suicides found significantly reduced numbers of third-order branches in the basilar dendritic arbor [81]. These data are the first evidence of altered cortical dendritic branching in a psychiatric disorder. As the proximal dendritic branches grow during perinatal development, and are generally less plastic at maturity than the more distal segments, this led the authors to speculate that differences in dendritic branching may reflect a biological predisposition to depression and suicide [81], or could result from perinatal stress exposure.

Notably, not only the glutamatergic pyramidal cells are affected by chronic stress, but also GABAergic interneurons undergo dendritic reorganization in the mPFC and dendritic hypertrophy was found in a subpopulation of interneurons identified as Martinotti cells [68]. Chronic adverse experiences further decrease GAD67 expression levels and the number of GAD67 expressing neurons [68, 185]. GAD67 is the 67 kDa isoform of glutamate decarboxylase enzyme which synthetizes GABA and is a marker for GABAergic neurons. Chronic stress also decreased the number of parvalbumin-positive interneurons in the PFC [185].

Not only PFC neurons are affected by chronic stress, but glial cells as well. Stress, e.g., inhibits gliogenesis in the PFC [39], results in impaired activity of microglia [86] and in a profound shortening of astrocytic branching and process length, as well as reduced GFAP expression [216]. These findings are in line with the astrocytic pathology in the PFC of depressed patients, that includes reduced astroglial cell numbers and related markers [167]. Finally, many of the cellular changes after stress can be hemisphere specific, and stress often abolishes or even reverses asymmetries at the cellular level [39, 40].

**Fig. 5:**
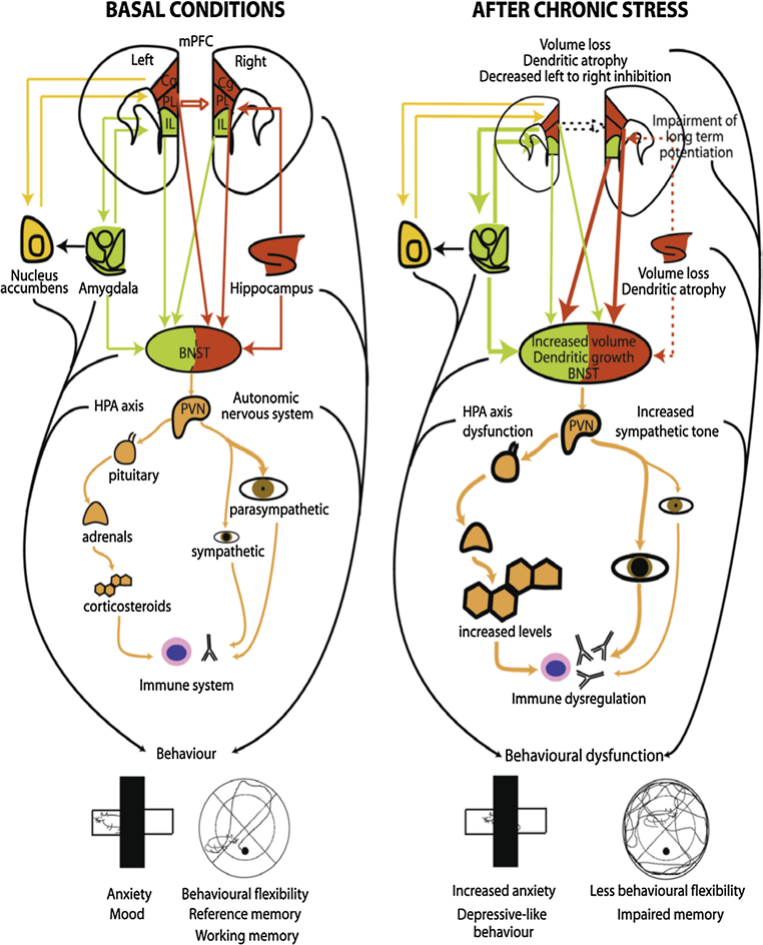
Schematic representation of the connections between the prefrontal cortex, the stress response and the immune system and the changed interactions during conditions of chronic stress. At basal conditions (*left panel*), the right medial prefrontal cortex (mPFC) is mainly under tonic inhibition from its left counterpart. Modulatory inputs from the mPFC, amygdala and hippocampus to the PVN relay on the bed nucleus of the stria terminalis (BNST). Furthermore, whereas activation of the infralimbic cortex (IL) and amygdala increases PVN activity, activation of the cingulate (Cg) and prelimbic (PL) parts of the PFC and from the hippocampus decreases it. In basal conditions the parasympathetic tone of the autonomic nervous system predominates. After chronic stress (*right panel*), which elevates glucocorticoid levels, changes are induced in the brain that include a decreased volume and dendritic retraction in the mPFC and hippocampus, but opposite changes in the BNST and amygdala (see Fig. 4). Damage to the hippocampus may decrease the influence of this brain structure on the mPFC and BNST (*dotted lines*); as a result, a reduced activity of the mPFC (especially in the *left* hemisphere) may occur, but an overactivation of the amygdala and over the neuroendocrine and autonomic control centers (BNST/hypothalamus). This may trigger HPA axis dysfunction, increase corticosteroid levels and activate the sympathetic nervous system, which, together, may induce immune dysregulation and contribute to behavioral dysfunction. Reproduced from [26], with permission

**Fig. 6:**
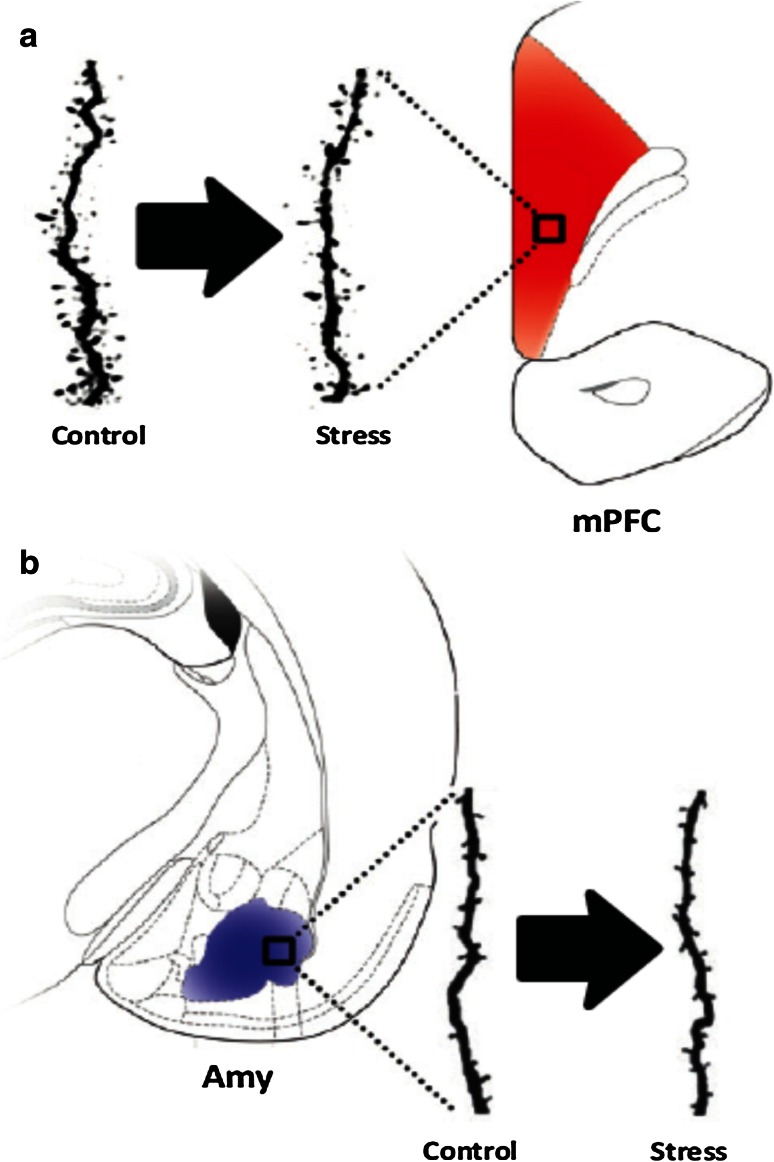
Scheme representing the contrasting effects of chronic stress on dendritic spine numbers in the prefrontal cortex (PFC) and amygdala. **a** A decrease in the number of spines occurs in pyramidal neurons of the infralimbic cortex of rats after repeated restraint stress (21 days). **b** By contrast, chronic immobilization stress (10 days) triggers an increase in the number of spines in basolateral amygdala spiny neurons in rats. *Amy* amygdala, *mPFC* medial prefrontal cortex. Reproduced, with permission, from [26]

Studies on the mPFC have been critical in showing that the impact of stress on one brain region can spread to other areas that are synaptically linked [200] involving, e.g., the corticostriatal network and indeed stress shifts decision-making to more habitual processing [50]. Thus, chronic stress-induced morphological and functional changes take place in the PFC and can result in various executive, cognitive and affective dysfunctions (Figs. 5, 6) (e.g., [18, 76, 114, 198]).

Effects of chronic stress on structure and function of the PFC are supported by human imaging studies. For example, significant reductions in the gray matter of DL–PFC and ACC were found in subjects with long-term occupational stress by MRI-based voxel-based morphometry [12]. Subjects reporting more uncontrollable and overwhelming stressors displayed blunted neural responses in mPFC following feedback processing as established by fMRI. In another fMRI study, patients with MDD and a history of childhood maltreatment showed reduced functional connectivity strength within the prefrontal-limbic-thalamic-cerebellar circuitry and these reductions were significantly correlated with measures of childhood neglect [230]. Maltreated children had volume reductions in the medial orbitofrontal cortex and middle temporal gyrus [44]. Similarly, early life stress in depressed patients decreased local connectivity of the ventrolateral PFC and correlated negatively with global connectivity for the DL–PFC [28].

In summary, there is a clear effect of early life stress or stress in adulthood on reductions in volume and alteration in functional connectivity of the PFC and related cortical limbic regions. Interestingly, these effects are present not only in depression but also in healthy controls who were exposed either to occupational or early life stress.

#### Inflammatory changes and depression

Although inflammatory changes alone may not typically initiate pathology, emerging evidence indicates that sustained inflammation can affect disease progression of various brain disorders, and is implicated in depression etiology [42, 218]. When activated, cells of the immune system produce pro-inflammatory cytokines that can induce behavioral withdrawal and activate the HPA axis [11]. Conversely, stress and/or elevated GC levels themselves are generally immunosuppressive and prevent the immune system from overshooting. Psychological stress is known to stimulate pro-inflammatory cytokine production in patients experiencing stress and anxiety, and elevations in interleukin-1β (IL-1β) and 6 (IL-6) and increases in macrophage activity have been reported in depression [42, 222]. Stress can provoke depression-like behaviors, mediated through the activation of inflammatory and anti-neurogenic mechanisms and pathways, some of which are even normalized by antidepressants [105]. Inflammation can further affect hippocampal function and cognition [138, 240, 246] and reducing it by anti-inflammatory drugs restored functional and structural plasticity in animal models [56, 138].

### Stress-related changes in PTSD

Post-traumatic stress disorder (PTSD) is a multisystem disorder with multiple comorbidities. PTSD patients have experienced severe, often life-threatening, episodes of stress that cause flashbacks, nightmares and sleep problems, emotional numbness or emotional outbursts, anhedonia, inappropriate startle reflexes and problems with memory and concentration [21]. Notably, the HPA changes in PTSD indicate low cortisol levels consistent with increased glucocorticoid sensitivity. In one particularly intriguing experimental protocol, 40 pairs of identical twins were studied, one of whom participated in the Vietnam war while the other stayed at home. Of those who experienced combat, 43 % developed PTSD. They turned out to have smaller hippocampi, but their stay-at-home twin brother did too. In contrast, those who did not develop PTSD had larger hippocampi, and so did their stay-at-home twin as well. A small hippocampus thus seems to be present already *prior to* the stressful experience and in fact to infer an increased vulnerability to PTSD [69, 180]. This goes back to the idea of either genetic factors, or life history and shaping events during critical development periods, or an interaction between the two, determining hippocampal volume early in life, and putting the individual on a trajectory for (psycho)pathology [30, 124, 161].

Thus, it remains elusive whether hypercortisolism is indeed responsible for hippocampal shrinkage, since combat-related PTSD is associated with decreased HPA axis activity and steroid feedback super-sensitivity that often lasts for decades after the initial trauma. It has been presumed that early on in the process the HPA axis may have been strongly activated. This is based on the observation that soldiers who had undergone random bombardments in the Korean war displayed markedly increased levels of cortisol, with the highest levels of cortisol in soldiers who had been in the greatest danger. It has therefore been hypothesized that high levels of cortisol at the time of the stressor would result in damage to the hippocampal neurons that may persist for many years after the original trauma and that could lead to reductions in hippocampal volume and subsequent differences in feedback or stress responsivity. However, those victims of rape or motor vehicle accidents who later developed PTSD appeared to have—already a few hours after the traumatic event—lower cortisol levels than victims who had no subsequent psychiatric disorder, or those who developed major depression. Yet, pituitary and adrenal hyperactivity to exogenous CRH and ACTH has been demonstrated in these patients. An increased sensitivity or up-regulation of GRs in PTSD and a pre-existing smaller hippocampal volume thus seems, at present, the best explanation [180, 239].

### Stress-related effects: relation to sex, aging, Alzheimer’s disease and Cushing’s disease

#### Gender differences in depression: relationship with HPA axis activity

Gender differences in stress regulation have important implications for understanding the physiological differences in the male and female brain and their impact on vulnerability to stress disorders. Women are generally better at expressing emotions, tend to score higher on emotional ratings on, e.g., neuroticism [73] and have an increased risk of suffering from mood disorders. Morphometrical studies have shown sexual dimorphisms in several brain structures implicated in emotional processing, including the cingulate and ventrolateral prefrontal cortices (larger in women) and the medial temporal structures, including the amygdala and BST (larger in men) that are implicated in emotional processing [70]. These structural differences are thought to be programmed by sex steroids during early development [70].

The possible importance of fluctuating levels of sex hormones as a risk factor for depression is underlined by the higher prevalence of premenstrual depression, antepartum or post-partum depression, and depression during the transition to menopause. Studies in rodents have further shown intrinsic differences in the way female and male brains respond to stress [113]. Human post-mortem brain, animal and cell-line studies confirm a key stimulating role of estrogens on CRH production, while androgens diminish CRH production [152]. These opposite effects on CRH neurons may underlie sex difference in the prevalence of depression.

#### Stress-related changes in Alzheimer’s disease (AD)

Stressful life experiences are implicated in sporadic forms of AD. A considerable portion of AD patients hypersecrete glucocorticoids or are non-suppressors of plasma cortisol following dexamethasone administration [145, 169, 208, 209]. Their GC levels generally correlate with their rates of cognitive impairment and the extent of neuronal remodeling [48, 89, 123]. The cognitive deficits in AD correlate primarily with hyperphosphorylated forms of the cytoskeletal protein tau, which, together with amyloid β (Aβ), has a pathogenic role in AD. Increased GC levels may not only induce hippocampal damage but can also potentiate Aβ toxicity [25, 91]. Conversely, dehydro-epiandrosterone (DHEA) and its sulphate (DHEAS) or GR blockade may exert a neuroprotective action [5, 141].

Some aging animals and mouse models of AD show changes in stress regulation too but also, in wild-type, middle-aged rats, chronic stress and GCs induce abnormal hyperphosphorylation of Tau in the hippocampus and PFC, with parallel impairments of hippocampus- and PFC-dependent behaviors. Exogenous GC application further potentiates the ability of centrally infused Aβ to induce hyperphosphorylation of Tau epitopes. Moreover, previous exposure to stress further aggravated the biochemical and behavioral effects of GCs in Aβ-infused animals [25, 193]. Thus, stress and GC exposure may have a cumulative impact on the onset and progression of AD pathology. Tau hyperphosphorylation may be instrumental in the negative effects of stress and GC on cognition, initially by increasing the production of pathogenic products of amyloid precursor protein (APP), followed by up-regulation of Tau kinases such as GSK3β and cdk5 and aberrantly hyperphosphorylated Tau [199].

#### Stress-related changes in Cushing’s syndrome

Cushing’s syndrome can occur after prolonged exposure to (tumor-derived) cortisol or synthetic steroids and usually induces some brain atrophy and cognitive dysfunction. Positive correlations exist between hippocampal volume and memory tests and negative ones with plasma cortisol levels. As at least partial recovery of brain shrinkage occurs after reversal of high GC exposure [19, 201] making massive, irreversible, cell loss unlikely [53, 75, 85, 242].

## Effects of stress on structural and molecular plasticity

Traditionally, affective and stress-related brain disorders were explained by neurochemical (mainly monoaminergic) imbalances. More recent studies indicate that impairments in structural plasticity and volumetric changes of specific limbic areas also contribute to their pathophysiology. Various candidate cellular substrates, like dendritic retraction, neuronal loss or glial changes have been proposed that are indeed all stress-sensitive [36, 127, 167]. Reciprocal relationships may exist between stress-related behaviors and changes in structural plasticity. Overall, it still remains unclear whether the above substrates should be classified as truly pathological or whether they may represent plastic and/or dynamic adaptations to a stressor that can to some extent be reversible.

### Dendritic remodeling

One of the best known forms of structural plasticity is dendritic retraction that was first observed in CA3 and CA1 hippocampal neurons following chronic stress exposure. At the structural level, prolonged exposure to high doses of corticosterone reduces apical (but not basal) dendritic complexity of CA3 pyramidal neurons [237] and a prolonged exposure to various types of chronic stress results in similar changes in different species [64, 129]. These changes in hippocampal dendritic morphology generally need several weeks to develop. In addition, chronic stress also leads to a loss of mossy fiber synapses, increased surface area of the post-synaptic density, and rearrangements of synaptic mitochondria and vesicles at the presynaptic terminals [176, 213].

### Spine density

The effects of stress on spine density are less clear. While increase in the number of spines were reported on CA3 pyramidal dendrites and in the size of the post-synaptic densities on CA1 synapses, others could not find changes in spine density [129] or reported a decrease in spines, that was notably rapidly reversible already after a recovery period or subsequent training [1, 176, 205]. Oscillating GC plasma concentrations along the circadian rhythm appear to be important for the elimination of spines present before motor learning, and also for the maintenance of new spines along with the retention of motor memory [115]. Spine elimination, but not their formation, was shown to require MR activation [115]. Together, these studies suggest that prolonged exposure to chronic stress and glucocorticoids markedly alters the number and morphology of both pre- and post-synaptic structural elements and thus strength of excitatory synapses in the hippocampus.

Dendritic spines are of particular relevance as they are critically involved in the storage of information and their density can be increased by stress under specific conditions [212]. Pyramidal cells of the hippocampus and PFC respond with reduced dendritic complexity and spine loss to chronic stress [71, 164], both of which are reversible following a recovery period [71, 165], but other regions appear resistant [194]. Consistent with circuit-specific effects of stress (Fig. 5), also increases were found in the dendritic arborization of orbital frontal cortex neurons, an effect opposite to what is observed in other cortical neuron populations. Interestingly, in the basolateral amygdala and nucleus accumbens (NAc), stress generally results in hypertrophy of dendritic arborization and increases in spine density (Fig. 6). In the amygdala, these changes do not normalize following a recovery period after stress [137, 225]. In addition to changes in spine density, chronic unpredictable stress reduces density of synapses in the rat PFC [94]. Similar reductions in synaptic density and a lower expression of synaptic function-related genes occur in the DL–PFC of MDD subjects [94].

### Apoptosis, neuronal death and neuropil loss

**Fig. 7:**
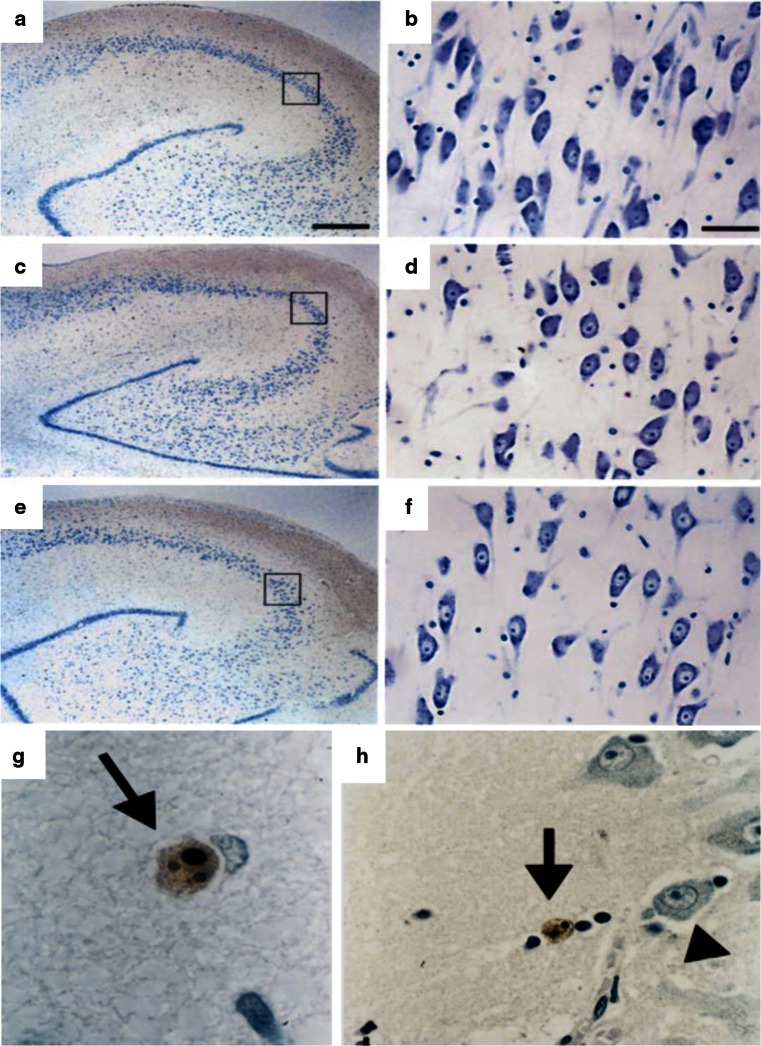
Photomicrographs of Nissl stained sections from the hippocampus of a depressed patient (**a**, **b**), a steroid-treated patient (**c**, **d**) and a control subject (**e**, **f**). **b**, **d,** **f** Shows the CA3 area of the same patients at higher magnification. Although some rare apoptotic cells (*brown* TUNEL-positive cells indicated by *arrows* in **g** and **h**, compared to intact, non stained neuronal nuclei nearby (*arrowhead*)) were seen outside of subregions predicted to be at risk for glucocorticoid overexposure like the dentate gyrus or entorhinal cortex, no morphological evidence for neuronal damage or massive cell loss was observed in any of the groups. *Bar* indicates 710 μm in (**a**) and 45 μm in (**b**). Reproduced, with permission, from [117, 142]

**Fig. 8:**
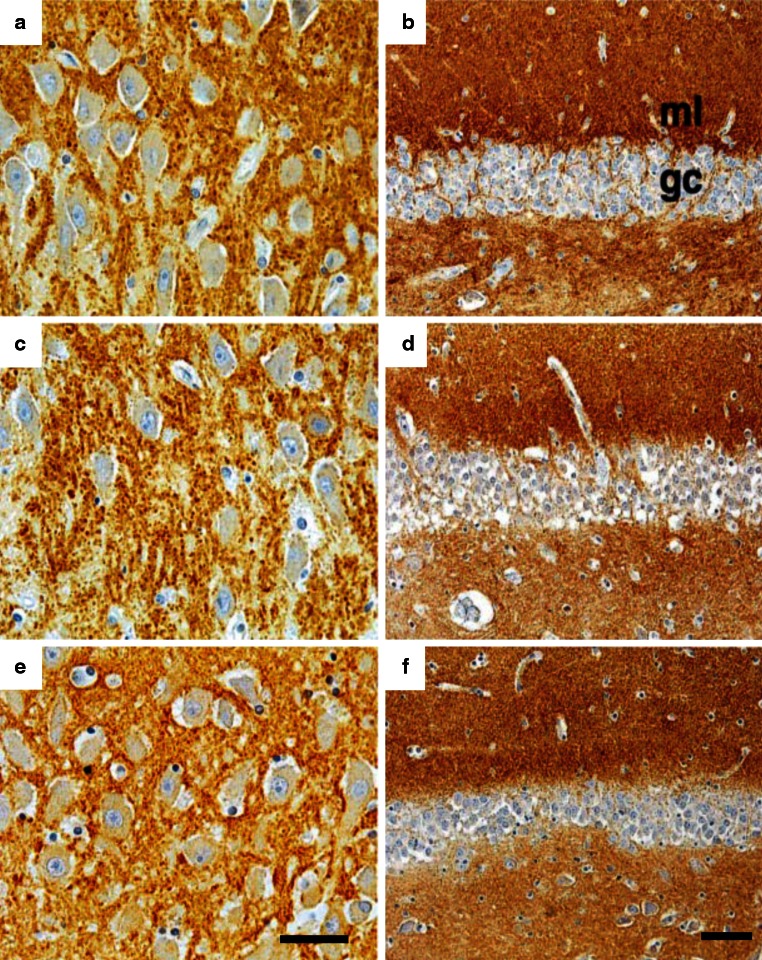
Immunohistochemical staining for synaptophysin (**a**, **c**, **e**) and for the neuronal growth-related phosphoprotein B-50 (**b**, **d**, **f**) in the hippocampus of **a**, **b** a depressed patient, **c**, **d** a steroid-treated patient and **e**, **f** a control subject. No marked qualitative difference is observed between the overall immunohistochemical staining patterns of the groups in the hippocampal subarea CA3 and the molecular layer of the dentate gyrus. Bar in **e** represents 50 μm, bar in **f** 115 μm. Reproduced, with permission, from [117, 142]

According to the glucocorticoid cascade hypothesis, neuronal apoptosis may underlie hippocampal volume shrinkage observed after stress [182]. Neuronal death has also been implicated in the cerebral shrinkage that occurs following prednisone administration and/or in the inflammatory changes in the cortex in depression [119, 134, 196, 209]. However, initial histological and neuropathological examination of the hippocampus of depression models or from patients that had been depressed or were exposed to synthetic corticosteroids, could not support this notion. In hippocampi from established depressed patients, no indications for obvious neuronal loss or for significant neuropathology could be found using a variety of relevant architectural, synaptic and glia markers [29, 117, 119, 142] (see Figs. 7, 8). In a very recent study, Boldrini et al. [17] reported fewer granule cells in the dentate gyrus of unmedicated depressed patients (without fewer neuronal progenitor cells) suggesting that cell maturation or turnover defects in this plastic hippocampal subregion might be related to the duration of the illness. Notably, the hippocampal volume reductions present in Cushing’s disease were reversed after cessation of steroid exposure [204]. Similar findings exist on ventricular enlargements that are reversible in certain conditions (e.g., following recovery from alcoholism or prolonged steroid use), thereby challenging the hypothesis that ventricular enlargement predicts neuronal loss [210]. This agrees with the general clinical experience with depressive or Cushing’s patients, in which treatment or operation can relieve their depressive symptoms, several of the HPA alterations, and even the hippocampal shrinkage, findings that would be hard to interpret had irreversible damage or massive cell loss been induced.

The incidence of apoptosis or cell loss in rodent stress models is rare and also seen after acute stress [79, 119, 241]. Chronic stress changed apoptosis in tree shrews only in specific hippocampal subareas and was normalized by tianeptine treatment [118]. These effects also occurred in associated cortical areas and were consistent with a general anti-apoptotic mode of antidepressant action [118, 134]. This bears considerable relevance for the interpretation of structural and/or neuropathological studies on the hippocampus in depression, where almost all patients generally receive antidepressant treatment [90, 117] and hence, effects of the disorder per se might be masked by the concomitant drug treatment. Yet, in drug-free, depressed patients [17], little differences were reported when compared to patients on treatment [17], and hippocampal volume reductions were then present as well.

### Adult hippocampal neurogenesis

**Fig. 9:**
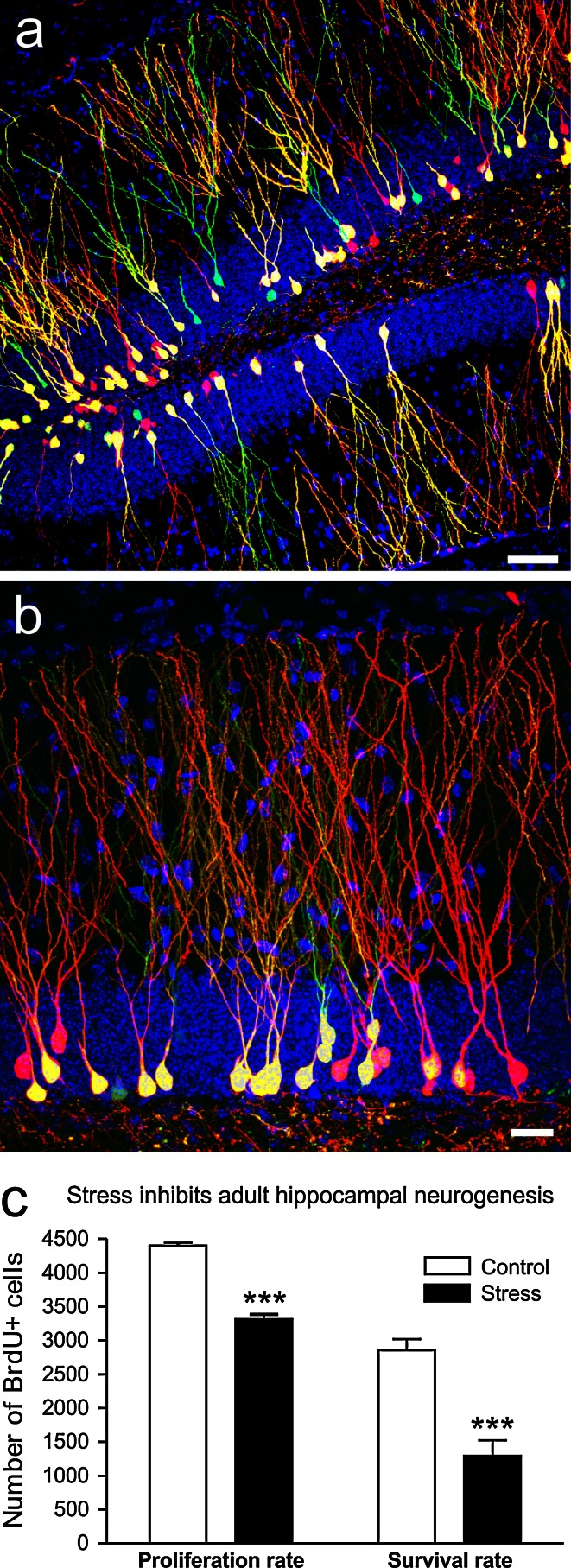
Chronic stress inhibits neurogenesis in the adult hippocampal dentate gyrus. Representative confocal images of newborn neurons in the hippocampus of adult mice with low (**a**) and high magnification (**b**). A mixture of retroviruses expressing *green* and *red* fluorescent protein (CAG-IRES-GFP and CAG-IRES-RFP) was injected into the dentate gyrus of adult mice to label the newly born cells. Double-transduced cells are in *yellow* and DAPI is in *blue*. B. Czéh, D. Refojo, and D.C. Lie unpublished observations. *Scale bars* **a** 50 μm, **b** 20 μm. **c** Chronic stress inhibits both the proliferation rate and the survival rate of the newly generated cells in the hippocampal dentate gyrus of adult rats as it was shown with BrdU-labeling in a chronic social defeat stress model (modified fom [39]). Data are mean ± SEM, group sizes *n* = 6 rats/group, ***p < 0.001. Similar changes are seen in depressed individuals [16, 121] and treatment with some, but not all, antidepressants can normalize these changes [16, 37, 120]

Another, relatively novel, form of neuroplasticity is neurogenesis that has been implicated in stress-induced hippocampal volume changes. Adult neurogenesis refers to the production of new neurons, an event that continues to occur in the adult brain of several mammalian species, including humans (Figs. 4, 9) [58, 120]. Neuron formation in the adult hippocampus received considerable attention during recent years and was proposed to contribute to depression etiology [96]. Although later studies suggested that neurogenesis was rather implicated in antidepressant drug actions [177], we still lack a coherent functional theory explaining how exactly newborn neurons in the hippocampus can contribute to mood, or to specific symptoms of depression, besides their cognitive deficits, which are related too, but not specific to mood disorders. Although a reduced rate of neurogenesis may reflect impaired hippocampal plasticity, reductions in adult neurogenesis alone are unlikely to produce depression. Lasting reductions in the turnover rate of DG granule cells, however, will alter the average age and overall composition of the DG cell population and thereby influence properties and vulnerability of the hippocampal circuit.

Furthermore, neurogenesis is regulated by exercise, inflammation and antidepressants (Fig. 4) [37, 120, 146, 177, 247] while stress potently inhibits neurogenesis in several species (Fig. 9) [120, 187]. Both psychosocial [74] and physical stressors [224] inhibit at least one or more phases of the neurogenesis process [74, 79]. Stress and GCs further interfere with most stages of neuronal renewal, proliferation, maturation and survival (Fig. 9) [79, 148, 187, 236].

The neurogenic hypothesis of depression proposes that prolonged reductions in neurogenesis, e.g., induced by stress, may affect hippocampal structure and volume in depression, and that successful antidepressant treatment would require increases in neurogenesis [96, 120]. In preclinical studies, the stress-induced suppression of DG neurogenesis can be prevented by antidepressant treatments, which can also have direct neurogenic effects in naive animals. They can block effects on depressive-like behavior and may also restore plasticity outside the hippocampus [24, 133, 146].

Inflammation also affects neurogenesis. Microglia are considered instrumental given their homeostatic role in inflammatory signalling that may become maladaptive in the chronically stressed brain [72, 86, 155]. Under physiological conditions, microglia exhibit a resting, ramified phenotype associated with the production of anti-inflammatory and neurotrophic factors, but when primed, e.g., by early life stress [51] or challenged by pathogens or damage during adult life, microglia can switch to an activated, amoeboid phenotype, that can initiate tissue repair, or rather produce cytokines that are detrimental for neuronal function and viability [52, 56]. Specific subsets of cytokines can be proneurogenic [10] while others decrease neurogenesis through IL-1 [247]. Pro-inflammatory mediators can further restrict neurogenesis [56, 247].

The exact underlying cellular mechanisms mediating the inhibitory effect of stress or inflammation on neurogenesis are largely unknown. Adrenal glucocorticoids have been suggested as key players and MR, GR and NMDA receptors have been identified on progenitor cells [74]. At the same time, several examples exist of a longlasting inhibition of neurogenesis after an initial stressor, despite later normalized GC levels [187]. This suggests that while glucocorticoids may be involved in the initial suppression of cell proliferation, particularly in early life, when neurogenesis is abundant, they are not always necessary for the maintenance of this effect [74].

Stress also affects levels of various neurotransmitters implicated in the regulation of neurogenesis: GABA [67], serotonin, noradrenalin, dopamine [6], cannabinoids, opioids and nitric oxide (see [6]). Stress further reduces the expression of several growth factors, such as brain-derived neurotrophic factor (BDNF), insulin-like growth factor-1 (IGF-1), nerve growth factor (NGF), epidermal growth factor (EGF), and vascular endothelial growth factor (VEGF), that all can influence neurogenesis (see, e.g., [186] while gonadal steroids should not be neglected either [63]. The proximity of the precursors to blood vessels further suggests a strong interaction with the vasculature and it is this population that is particularly sensitive to stress [80].

Although there is extensive preclinical evidence that antidepressants affect hippocampal neuroplasticity, information regarding the possible impact of medication on hippocampal volume in MRI studies of MDD patients is limited. A recent study by Huang et al. [90], found smaller DG volumes in unmedicated depressed patients and a post-mortem analysis reported the same [17] which would be consistent with the neurogenic hypothesis of depression. Interestingly, both subfield and posterior hippocampal volume reductions were reported that were only seen in unmedicated depression but were absent in patients treated with antidepressants. Although it is so far not possible to reliably detect ongoing neurogenesis in vivo [35, 131], these data are consistent with preclinical studies demonstrating subregional specific effects of stress and its modulators on neurogenesis, and/or on hippocampal functionality [99].

While neurogenesis in the mature human hippocampus is a rare event [14, 58, 101], changes have been reported following antidepressant treatment in middle aged, but not older depressed patients [15, 16, 121]. In a recent post-mortem study of major depressed patients, the volume of the histologically defined dentate gyrus was in fact 68 % larger in SSRI-treated depressed subjects [16]. SSRI treatment also substantially increased neural progenitor cells (NPCs) in the dentate gyrus [15], although this was not replicated by others [170] and may depend on the age of the patients [121].

### The glutamatergic and GABAergic systems in depression

The glutamatergic pathway shows alterations in patients with mood disorder [9, 174]. Particularly, since glutamate stimulates the HPA axis antiglutamatergic agents, like ketamine [95, 244] are considered promising targeta for new antidepressants. In patients with MDD, glutamine synthetase (GS) transcripts were down-regulated in the anterior cingulate cortex and dorsolateral PFC [27]. The expression of glutamate transporters, the excitatory amino acid transporters (EAAT), EAAT1, EAAT2 are reduced frontal brain regions in MDD [27]. Accumulations of extracellular glutamate may not only perturb the ratio of excitatory–inhibitory neurotransmitters [34], but could also damage neurons and glia.

Regarding the GABAergic system, the majority of studies revealed lower GABA levels in the brain of depressed patients, suggesting a hypoactive GABAergic neurotransmission, including the PVN [9, 126]. GABA also inhibits the HPA axis in the hypothalamus [103] and GABAergic drugs can help maintain a positive response during antidepressant treatment [126].

### Glial plasticity

During recent years, also glial abnormalities have been implicated in the pathophysiology of mood disorders. Glial cells slightly outnumber neurons in the human hippocampus and constitute a substantial volume fraction. During pathophysiological conditions, specific glial subtypes may become activated, or may die, or, similarly to the dendritic debranching documented in neurons, may retract their elaborate branching processes, and thereby reduce hippocampal volume. A considerable proportion of the astrocytes further express GRs in the rodent [66] and human hippocampus [231] and similar to neurons, these cells are stress responsive too. Typically, astrocytes are identified by their GFAP (glial fibrillary acidic protein) expression and stress modulates GFAP expression in rodents: while acute physical stress increase GFAP immunoreactivity in several brain regions [106], chronic stress reduced GFAP mRNA and protein expression levels in the prefrontal cortex and hippocampus [3]. As corticosterone lowers GFAP levels in rat brain [144], elevated glucocorticoids are likely instrumental in these effects.

Further studies demonstrated that in laboratory animals, exposure to long-term stress, either during early life or adulthood, decreased number and somal volume of GFAP-positive astrocytes in the hippocampus and in several other stress-related brain areas [38, 111]. This implied that chronic stress may cause astrocytic loss, while more recent experiments did not substantiate that [216]. For example, in vitro experiments showed that dexamethasone treatment of astrocytes, derived from hippocampal primary cell cultures, results in growth inhibition and moderate activation of caspase 3, which is not followed by apoptosis [241], suggesting that hippocampal astrocytes are resistant to glucocorticoid-induced apoptosis.

A recent in vivo study suggests that the reduced number of GFAP-positive cells after stress may not reflect cell death. By comparing the results of different labeling methods, it was found that chronic stress was associated with a decrease in GFAP-+ cell numbers, but there was no indication for astrocytic cell loss based on Nissl staining or S100ß-immunoreactivity [216]. This latter study also showed that astrocytes respond to chronic stress by reorganizing their cellular morphology and reducing the length, complexity and volume of their processes [216].

Chronic stress can also reduce the proliferation rate of glial cells. This was shown in the medial prefrontal cortex of rats subjected to 5 weeks of social defeat, or to chronic unpredictable stress or after chronic corticosterone administration [7, 39]. Similarly, prolonged and elevated glucocorticoid treatment inhibits NG2-positive cell proliferation in the adult rat hippocampus [144] reflecting changes in oligodendrocyte precursors or in a distinct mature glial type. Chronic stress also promotes significant structural remodeling of microglia, and can enhance the release of pro-inflammatory cytokines from microglia [227].

Finally, glial cells, especially astrocytes, are key components of the “neurogenic niche” that provides the necessary local microenvironment for generation of neurons in specific brain areas. They support maturation and integration of newborn neurons, both physically and by releasing a cocktail of growth factors and cytokines. Together, this implies that GCs not only are influenced by stress, but also stimulate interactions between astrocytes and neural progenitors.

In humans, post-mortem analysis of tissue from depressed patients has revealed reductions in glial numbers in the amygdala and prefrontal, orbitofrontal and cingulate cortices [20, 32, 149]. Accumulating data demonstrates that not only astrocytic cell numbers are reduced in depressed patients, but several typical structural and functional astrocytic markers as well, like GFAP, gap junction proteins, the water channel aquaporin-4 (AQP4), a calcium-binding protein S100B and glutamatergic markers including the excitatory amino acid transporters 1 and 2 (EAAT1, EAAT2) and glutamine synthetase [167].

In contrast, studies on post-mortem hippocampal samples so far failed to find significant reductions in neuron or glial cell numbers, while confirming the volume reduction in depressed patients [29]. One should add that besides astrocytes and oligondendrocytes, also microglia have also been implicated in pathological mood regulation [55, 60]. In vivo imaging studies in depressed patients demonstrate white matter abnormalities suggesting a contribution of oligodendrocytes to MDD etiology. MRI and also diffusion tensor imaging studies revealed white matter hyperintensities particularly in elderly subjects with late-life depression (reviewed in [55]) which was substantiated by post-mortem data reporting lower density of oligodendrocytes in the MDD brain and a reduced expression of oligodendrocyte-specific gene transcripts [55]. Animal models based on chronic stress paradigms also showed cellular and molecular changes in limbic structures that indicate an involvement of oligodendrocytes [39, 43, 207].

### Water content, volume changes and altered vasculature

The controversial findings on hippocampal volume decrease without significant cell loss might also be explained by shifts in water content and the volumetric reduction of limbic structures is indeed often accompanied by enlarged cerebral ventricles [97]. In support, significantly shortened T1 relaxation times were found for the hippocampus, especially in elderly depressed patients, indicative of differences in hippocampal water content. A recent study used multimodal in vivo imaging, incorporating structural magnetic resonance imaging (MRI), and MR spectroscopy (^1^H-MRS) in rats exposed to ethanol [242]. While MRI revealed expansion of ventricles, volume changes in dorsal or ventral hippocampi, caudate or thalamus were not detected. Also, all MR parameters returned to baseline with 7 days of recovery [242]. Thus, the rapid recovery of ventricular volume and the absence of detectable volume reductions in brain regions adjacent to the ventricles argue against neuronal or tissue atrophy as a mechanism to explain ventricular expansion but rather suggest lower tissue water content. Thus, a rapid fluid redistribution may be followed by compensatory ventricular volume changes in stress-related psychopathologies.

Water homeostasis is largely regulated by aquaporin water channels. In the CNS, their major representative is AQP4 which is expressed in the end-feet of astrocytic processes, but not in neurons [211]. In the human orbitofrontal cortex (Brodmann’s area 47; gray matter), the density of AQP4 immunoreactive astrocytic end-feet was reduced by 50 % in patients with major depression [168] while the coverage of vessels by GFAP-immunoreactive processes did not differ from controls. These data indicate possible disturbances in water homeostasis, at least in this brain region.

Finally, a recent study revealed that stress reduced numbers of microvessels and capillarization in the hippocampi of stressed rats [41, 80] which coincides with clinical studies and metareviews indicating that increased psychological distress and depression are associated with increased stroke risk and mortality [150].

## Conclusions

Exposure to chronic or severe stress has profound effects on the structural and functional integrity of limbic brain areas that not only coordinate the stress response, but are also exposed to the altered expression levels of different hormones, neurotransmitters and trophic factors. The central role of the HPA axis in these events has been most thoroughly investigated. Current experimental, post-mortem and in vivo imaging techniques have revealed various subtle morphological changes detectable both at the cellular level (affecting spines, dendrites, endothelial cells of the vasculature and glial and neuronal cell numbers), and in the gross morphology of specific brain areas (MRI findings). These changes thus appear to affect almost all cellular components of the CNS and many efforts are now undertaken to uncover the exact molecular mechanisms. While earlier studies highlighted disturbances in the monoaminergic systems, the glutamatergic and GABAergic systems receive attention too.

Most of the cellular responses to stress are initially plastic in nature and can normalize following appropriate recovery periods. These changes, particularly during the early phase, are often adaptive and essential for successful coping with stress. It is still very difficult to pinpoint when adaptive changes turn into maladaptive and/or pathological, and when systems start to deteriorate. Notably, many stress-induced morphological changes are specific to selected brain areas, and even to specific cell types, where they often correlate well with the functional disturbances of that given brain structure.

Finally, in humans, severe or repeated stress often contributes to the development, or can worsen the outcome, of psychopathology. Here, it is difficult to separate initial structural and/or neuropathological changes specific to the disease from the ones that result from the (additional) stress exposure per se.

## Abbreviations

Aβ: Amyloid beta (β) protein
ACC: Anterior cingulate cortex
ACTH: Adrenocorticotropic hormone
AD: Alzheimer’s disease
ANS: Autonomic nervous system
APP: Amyloid precursor protein
AQP4: Water channel aquaporin-4
AR: Androgen receptor
AVP: Arginine vasopressin
BD: Bipolar depression
BDNF: Brain-derived neurotrophic factor
BLA: Basolateral amygdala
BST: Bed nucleus of the stria terminalis
CBG: Glucocorticoid binding globulins
CDK5: Cyclin-dependent kinase 5 (cell division protein kinase 5)
CRH: Corticotropin-releasing hormone
CRH-R: Corticotropin-releasing hormone receptor
DHEA: Dehydro-epi-androsterone
DHEAS: Dehydro-epi-androsterone sulphate
DL–PFC: Dorsolateral prefrontal cortex
EAAT: Excitatory amino acid transporters
EGF: Epidermal growth factor
ER-α: Estrogen receptor-alpha
GAD: Glutamic acid decarboxylase
GC: Glucocorticoid hormone
GFAP: Glial fibrillary acidic protein
GR: Glucocorticoid receptor
GS: Glutamine synthetase
fMRI: Functional magnetic resonance imaging
H-MRS: Proton magnetic resonance spectroscopy
HPA: Hypothalamic–pituitary–adrenal
IL-1β: Interleukin 1-beta
IGF-1: Insulin-like growth factor-1
LTD: Long-term depression
LTP: Long-term potentiation
MDD: Major depressive disorder
mPFC: Medial prefrontal cortex
MR: Mineralocorticoid receptor
MRI: Magnetic resonance imaging
NAc: Nucleus accumbens
NGF: Nerve growth factor
NPCs: Neural progenitor cells
OXT: Oxytocin
pgACC: Pregenual anterior cingulate cortex
PFC: Prefrontal cortex
PSD-95: Post-synaptic density-95
PTSD: Post-traumatic stress disorder
PVN: Paraventricular nucleus of the hypothalamus
SNPs: Single-nucleotide polymorphisms
SSRI: Selective serotonin reuptake inhibitor
SVZ: Subventricular zone
UD: Unipolar depression
VEGF: Vascular endothelial growth factor
